# Detecting Service Chains and Feature Interactions in Sensor-Driven Home Network Services

**DOI:** 10.3390/s120708447

**Published:** 2012-06-25

**Authors:** Takuya Inada, Hiroshi Igaki, Kosuke Ikegami, Shinsuke Matsumoto, Masahide Nakamura, Shinji Kusumoto

**Affiliations:** 1 Graduate School of System Informatics, Kobe University, 1-1 Rokkodai-cho, Nada-ku, Kobe, Hyogo 657-8510, Japan; E-Mails: inada@ws.cs.kobe-u.ac.jp (T.I.); ikegami@ws.cs.kobe-u.ac.jp (K.I.); shinsuke@cs.kobe-u.ac.jp (S.M.); masa-n@cs.kobe-u.ac.jp (M.N.); 2 Graduate School of Information Science and Technology, Osaka University, 1-5 Yamadaoka- Suita, Osaka 565-0871, Japan; E-Mail: kusumoto@ist.osaka-u.ac.jp

**Keywords:** smart home, home network system, sensor-driven service, feature interactions, detection, validation

## Abstract

Sensor-driven services often cause chain reactions, since one service may generate an environmental impact that automatically triggers another service. We first propose a framework that can formalize and detect such service chains based on ECA (event, condition, action) rules. Although the service chain can be a major source of feature interactions, not all service chains lead to harmful interactions. Therefore, we then propose a method that identifies feature interactions within the service chains. Specifically, we characterize the degree of deviation of every service chain by evaluating the gap between expected and actual service states. An experimental evaluation demonstrates that the proposed method successfully detects 11 service chains and 6 feature interactions within 7 practical sensor-driven services.

## 1. Introduction

Research and development of *home network systems (HNS)* draws great attention as a key technology for the smart homes [1,2]. In the HNS, household appliances (e.g., TV, DVD Recorder, air-conditioner, lamp, fan) and equipments (e.g., curtain, ventilator, ceiling light) are integrated via a network to achieve various value-added services. Moreover, introducing sensors (e.g., temperature, brightness, motion, electricity, touch) to the HNS can provide autonomous *sensor-driven services*. A sensor-driven service is triggered automatically, depending on a designated *context* characterized by sensor values. Examples of the sensor-driven services are listed as follows [3]:

**DVD Theater Service (DVD-T):** This service allows a user to watch a movie in a theater-like atmosphere. When the user touches a touch sensor, a TV is turned on, a light is dimmed, a curtain is closed, and a DVD recorder is played.
**Automatic Light Control Service (ALC):** This service automatically turns on a light when the room becomes dark. When the brightness is lower than 200 lx and the user is in the room, a light is automatically turned on.
**Automatic Room Heating Service (ARH):** This service automatically heats the room with an air-conditioner. When the room temperature is colder than 14 °C, the air-conditioner is turned on with a heating mode of 28 °C.
**Energy Saving Air-Conditioning Service (ESAC):** This service controls an air-conditioner for energy saving. When the total electricity exceeds 1,500 Wm and the air-conditioner is working in a heating mode, the temperature of the air-conditioner is adjusted to 25 °C.

These sensor-driven services work fine when they are used separately. However, when multiple services are deployed in the same environment, execution of a service successively triggers another service in a certain condition. We define this phenomena as *service chain*, representing a chain reaction of sensor-driven services within the HNS. The service chain often causes undesirable conflicts among services, known as the *feature interaction problem* [3,4]. For the above four services, we can observe the following service chains.

**Service Chain C1 (DVD-T⇒ALC):** When a user runs DVD-T, a light is diminished and the room becomes dark. As a result, the condition “brightness is lower than 200 lx” holds. So, ALC is triggered and a light is turned on. This ruins the theater-like atmosphere of DVD-T.
**Service Chain C2 (ARH⇒ESAC):** Suppose that the room temperature drops below 14 °C, and that ARH starts the air-conditioner with the temperature setting 28 °C to heat the room. In course of time, the electricity consumption rises greater than 1,500 Wm. As a result, ESAC is triggered and the temperature setting is adjusted to 25 °C. Thus, the temperature setting of ARH is overwritten by ESAC.
**Service Chain C3 (ALC⇒ESAC):** Suppose that room brightness drops below 200 lx, and that ALC turns on a light. If the total electricity becomes greater than 1,500 Wm at this time, ESAC is automatically triggered.

In the above scenarios, the service chains C1 and C2 cause undesirable or unexpected behaviors for the users, whereas the service chain C3 causes nothing wrong. Thus, not all service chains are harmful, but they are potential factors of feature interactions. So, when deploying many sensor-driven services in the HNS, all service chains should be identified in advance. Furthermore, it is also important to extract harmful service chains automatically.

The goal of this paper is to propose a framework that can characterize and detect all potential service chains and feature interactions for given sensor-driven services within the HNS. Specifically, we first introduce the *ECA (event, condition, action) rules* to describe the sensor-driven services. We then develop the *environment effect model* to capture explicitly how each appliance affects the environment. Finally, we develop the *service chain detection algorithm* and *feature interaction detection algorithm*. The service chain detection algorithm can derive concrete conditions describing when the service chain occurs. In the feature interaction detection algorithm, we propose a severity evaluation method of each service chain by comparison between an expected state by a sensor-driven service and an actual state by a service chain including the sensor-driven service.

We conducted a case study with 7 practical services operated in our HNS. As a result, we could detect 11 service chains, in which 6 harmful feature interactions are identified.

## 2. Preliminaries

### 2.1. Home Network System

A home network system (HNS) consists of multiple *networked appliances* connected to the local area network at home. Each networked appliance has control APIs, with which users or software agents can control the appliance via the network. Each appliance is generally equipped with a network adapter, a processor and a storage.

A HNS typically involves a *home server*, which manages all the appliances deployed. It also plays a role of *gateway* to the external network. More importantly, various value-added *services* are installed in the home server. When a service is requested, the home server executes APIs of the appliances according to the logic specified in the service. As seen in Section 1, a service can orchestrate different appliances at the same time. In our research group, we are building an actual HNS (CS27-HNS) using legacy home appliances [5].

### 2.2. Sensor-Driven Service in HNS

Introducing sensors to the HNS can make the services autonomous and context-aware. Since the sensors can capture events and contexts, we use them as triggers of the service. In this paper, we write *sensor-driven service* to represent any HNS service triggered by a sensor. For example, let us take Automatic Light Control Service (ALC) in Section 1. The condition “the brightness is lower than 200 lx” can be evaluated by a light sensor. Thus, ALC can be implemented as a program which invokes API Light.on() when the reading of the light sensor becomes less than 200.

The sensor-driven services are smart and convenient in the sense that they do not require human operations. However, if many services are deployed in the same environment, they often cause unexpected service chains, as seen in Section 1. Since the number of potential service chains grows combinatorially in the number of services, we need a systematic method to detect and resolve the service chains.

### 2.3. Previous Work: Feature Interactions in HNS

In our previous work [3,4], we proposed an object-oriented modeling method to formalize and detect feature interactions in the HNS. In this method, we modeled every appliance (or an environment) as an *object* with *properties* and *methods.* The properties characterize the internal state of the appliance (or the environment), while the methods correspond to the API. Also, we defined every service as a sequence *m*_1_(); *m*_2_(); …; *m_n_*() of the appliance methods. Then, we defined that a feature interaction between services *s*_1_ and *s*_2_ occurs if a method *m*() of *s*_1_ and another method *m*′() of *s*_2_ conflict, either locally on an appliance object (called, *appliance interaction*) or indirectly via an environment object (called, *environment interaction*). However, this method assumed that every service is triggered *manually* by the user. Thus, the sensor-driven services and the incidental service chains were beyond the scope.

## 3. Modeling Sensor-Driven Services and Service Chains

### 3.1. Describing Sensor-Driven Services with ECA Rules

In order to capture the nature of the sensor-driven services, we here introduce the *ECA (event, condition, action) rules* [6] for the service description. A *sensor-driven service S* is defined by *S* = (*E_S_, C_S_, A_S_*) where

- *E_S_* is an *event* that triggers *S*, which is defined by a condition over a *single* environment property.
- *C_S_* is an *enabling condition* to determine the execution of *S*. On detecting *E_S_, S* is actually executed only when *C_S_* is satisfied. *C_S_* is defined by a condition over appliance properties and environment properties.
- *A_S_* is an *action* to be executed, which is defined by a sequence *m*_1_(); *m*_2_(); …; *m_n_*() of appliance methods.

This model newly involves the event and condition, compared to the one in the previous work.

**Figure 1.**
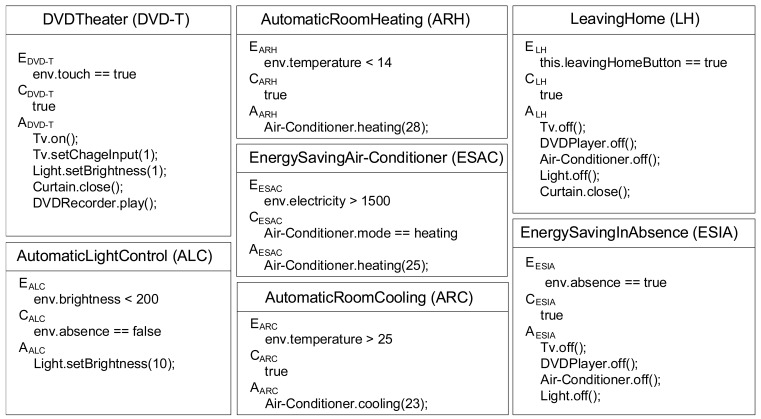
Service description of sensor-driven services with ECA rules.

Figure 1 shows service descriptions of seven sensor-driven services, including the 4 services seen in Section 1 and 3 more described below.

**Automatic Room Cooling (ARC):** This service automatically cools down the room, using an air-conditioner. When the room temperature is warmer than 25 °C, the air-conditioner is turned on with a cooling mode of 23 °C.
**Leaving Home (LH):** This service shuts down all appliances when a user leaves home. When the user touches a button in the entrance, a TV, a DVD player, an air-conditioner and a light are turned off, and a curtain is closed.
**Energy Saving in Absence (ESIA):** This service automatically turns off appliances for energy saving in user's absence. When a sensor detects that nobody is in the room, the service turns off a TV, a DVD player, an air-conditioner and a light.

In Figure 1, env denotes a prefix of an environment property. Each event (or condition) is supposed to be detected (or evaluated, respectively) by appropriate sensors in the HNS. In each action, A.m() denotes a method m() of an appliance A. Let us take the description of ALC. The event of ALC is env.brightness < 200, specifying that the service is triggered when the brightness is less that 200 lx. The condition env.absence == false means that the service should be enabled only when somebody is in the room. If ALC is executed, the light is turned on with brightness level 10 as specified in the action Light.setBrightness(10).

### 3.2. Modeling Environmental Effects of Appliances

To detect the service chains, we have to know how much *effect* is given to the environment as a result of a service. Such effect is produced by appliance methods executed as an action of the service. For example, Light.on() increases env.brightness, and Air-Conditioner.cooling() decreases env.temperature. Therefore, we propose an *environment effect model* for each appliance, to define explicitly how the appliance gives the environmental effects.

As shown in Section 2.3, every appliance can be regarded as an object with internal states. Therefore, we model every appliance as an FSM (finite state machine) specifying the effects within transitions. Let *d* be an appliance. The *environment effect model* of *d* is defined by an FSM *EM_d_* = (*S_d_, M_d_, T_d_, s*_0_, *e_d_*), where

- *S_d_* is a set of states of *d*.
- *M_d_* is a set of appliance methods of *d*.
- *T_d_* : *S_d_* × *M_d_* → *S_d_* is a state transition function.
- *s*_0_ ∈ *S_d_* is the initial state.
- *e_d_* is an environment effect function, associating each transition *t* ∈ *T_d_* with a set of expressions over environment properties.

Tables 1 shows the environment effect models of an air-conditioner, a TV and a light, respectively. Each table describes an FSM in a table form, where a row represent a state, a column represents a method. Each entry represents a state transition, containing the effects to the environment and the next state (labeled by next). In the effects, =, += and −= respectively represent substitution, addition and subtraction operators. For example, Table 1(b) represents that the TV has two states OFF and ON. If method on() is executed within OFF, the state moves to ON. At this time, as the environment effects, the electricity is increased by 500 Wm, and the brightness is increased by 200 lx.

#### Assumption 1

Effects of every appliance to environmental values by each appliance are reflected instantly.

For some environment properties, the effect may be given gradually For example, it takes time for an air-conditioner to change the room temperature in the designated value. However, in this paper we define the environment effect by an expected value converged after sufficient time.

We assume that every appliance *d* in the HNS is first in the initial state of *EM_d_*. When a service *S* = (*E_S_, C_S_, A_S_*) is executed, each method *d.m*() in *A_S_* is executed one by one. Then, a corresponding transition *t* in *EM_d_* occurs and environment effects *e*(*t*) are accumulated to the environment.

### 3.3. Service Chain

A service chain occurs when the result of one service triggers another service, successively. We try to formalize this mechanism using the proposed service description and the environment effect model. Let *S_A_* = (*E_S_A__, C_S_A__, A_S_A__*) and *S_B_* = (*E_S_B__, C_S_B__, A_S_B__*) be two services. A *service chain* from *S_A_* to *S_B_*, denoted by *S_A_* ⇒ *S_B_*, occurs when the environment effects produced in *A_S_A__* create an environment (state) where *E_S_B__* is satisfied. A chain *S_A_* ⇒ *S_B_* may cause another chain *S_B_* ⇒ *S_C_*, creating *S_A_* ⇒ *S_B_* ⇒ *S_C_*. Also, if *S_B_* triggers *S_A_* again, then a loop *S_A_* ⇒ *S_B_* ⇒ *S_A_* ⇒ *S_B_* ⇒ … may be produced.

Note that the occurrence of *S_A_* ⇒ *S_B_* is *conditional*. That is, it depends on the current states of the appliances and the environment. For example, for ALC⇒ESAC in Section 1, if no other appliance is working before the execution of ALC, ESAC may not start. Therefore, it is important to identify the *pre-condition* of the service chain, explaining when the chain occurs. We denote [*cond*]*S_A_* ⇒ *S_B_* to represent that a chain *S_A_* ⇒ *S_B_* occurs when a pre-condition *cond* holds.

### 3.4. Feature Interactions in Service Chain

Service Chain may cause feature interactions. However, all service chains do not lead to harmful feature interactions. In the chain scenarios described in section D, scenario C1 is considered as harmful one. In the scenario C1, service chain DVD-T-ALC prevents a user from watching a movie in a theater-like atmosphere contained in the requirement of DVD-T. On the other hand, in the chain scenario C2, ESAC makes preset temperature of the air-conditioner lower than a user's requirement in ARH. Although this scenario is also considered as a feature interaction, a user may accept the change of room temperature for 1 degree. Moreover, energy saving, which is one of the requirements in ESAC, is satisfied in the scenario.

The service chain causes various feature interactions which can be serious or acceptable. Then, we propose a method to evaluate the severity of feature interactions.

## 4. Detecting Service Chains among Sensor-Driven Services

### 4.1. Service Chains Detection Algorithm

Now we present an algorithm that can automatically detect the service chains among sensor driven services, using the proposed service description and the environment effect model. Specifically, for a given pair of services *S_A_* = (*E_S_A__, C_S_A__, A_S_A__*) and *S_B_* = (*E_S_B__, C_S_B__, A_S_B__*), the algorithm checks if *S_A_* ⇒ *S_B_* occurs. Moreover, if the chain occurs, the algorithm derives a concrete pre-condition *cond* such that [*cond*]*S_A_* ⇒ *S_B_* holds. Intuitively, the algorithm checks if the environment effects produced by *A_S_A__* may create a situation where *E_S_B__* is satisfied, as discussed in Section 3.3. It consists of the following nine steps.

**Step 1:** Pick out an environment property *p* in *E_S_B__*.
**Step 2:** By analyzing *A_S_A__*, obtain a set *M_p_* = {*m*_1_(), *m*_2_(), …, *m_n_*()} ⊆ *A_S_A__* of appliance methods where every *m_i_*() has an environment effect on *p*.
**Step 3:** For every *m_i_*() ∈ *M_p_*, analyze the environment effect models and obtain a set *T_m_i__* of any state transitions on which *m_i_*() appears.
**Step 4:** Construct a Cartesian product *T_p_* = *T*_*m*_1__ × *T*_*m*_2__ × … × *T_m_n__*. Each element *tt* = (*t*_1_, *t*_2_, …, *t_n_*) ∈ *T_p_* represents a sequence of transitions each of which has an environment effect on *p*.
**Step 5:** For each *tt* = (*t*_1_, *t*_2_, …, *t_n_*) ∈ *T_p_*, calculate the *total environment effect TE*(*tt*) = Σ [*e_p_*(*t*_1_), *e_p_*(*t*_2_), …, *e_p_*(*t_n_*)], where *e_p_*(*t_i_*) is an environment effect added to the environment property *p* by the transition *t_i_*. The semantics of Σ will be defined later.
**Step 6:** Let *s_e_* be any environment state where *S_B_* is enabled (i.e., *E_S_B__* holds), and let *s_d_* be any state where *S_B_* is disabled (i.e., ¬*E_S_B__* holds). For each *tt* ∈ *T_p_*, if there exists such a pair (*s_d_, s_e_*) that *TE*(*tt*) changes the state *s_d_* into *s_e_*, then detect a service chain *S_A_* ⇒ *S_B_*. For this, we call *tt potential transition sequence of the service chain*.
**Step 7:** If *tt* = (*t*_1_, *t*_2_, …, *t_n_*) ∈ *T_p_* is a potential transition sequence of the service chain, for each *t_i_* (1 ≤ *i* ≤ *n*), derive a condition *dcond_i_* of an appliance where *t_i_* is executed. Then, make a conjunction *dcond_p_* = *dcond*_1_ ∧ *dcond*_2_ ∧ … ∧ *dcond_n_*, which is a pre-condition of the service chain w.r.t. the appliances. In addition, obtain a condition *econd_p_* on the environment where *s_d_* of Step 6 is satisfied, which is a pre-condition of the service chain w.r.t. the environment.
**Step 8:** Validate that *cond_p_* = *dcond_p_* ∧ *econd_p_* does not contradict to *E_S_A__* ∧ *C_S_A__*. If there is no contradiction, derive *cond_p_* as the pre-condition of *S_A_* ⇒ *S_B_*. If there is a contradiction, *S_A_* is disabled. So conclude that *S_A_* ⇒ *S_B_* does not occur. (End)

Let *v_i_* be the value of the environment effect *e_p_*(*t_i_*). The semantics of Σ in Step 5 is defined by one of the following S1, S2 or S3, depending on the environment property *p* interested.

**S1(Arithmetic Sum):** Σ [*e_p_*(*t*_1_), *e_p_*(*t*_2_), …, *e_p_*(*t_n_*)] = *v*_1_ + *v*_2_ + … + *v_n_*. This semantics is taken when *p* is a *cumulative* property, including brightness, sound volume, *etc.*
**S2(Maximum Value):** Σ [*e_p_*(*t*_1_), *e_p_*(*t*_2_), …, *e_p_*(*t_n_*)] = *max*(*v*_1_, *v*_2_, …, *v_n_*). This semantics is taken when the value of *p* is overwritten by the maximum one, including the temperature of heater, wind level, *etc.*
**S3(Minimum Value):** Σ [*e_p_*(*t*_1_), *e_p_*(*t*_2_), …, *e_p_*(*t_n_*)] = *min*(*v*_1_, *v*_2_, …, *v_n_*). This semantics is taken when the value of *p* is converged to the minimum one, including the temperature of air-conditioner.

We briefly summarize the purpose of each step of the algorithm. Step 1 finds the key property *p* by which the service chain *S_A_* ⇒ *S_B_* may occur. Step 2 extracts appliance methods that potentially give an effect to *p*. Step 3 identifies all state transitions related to the methods within the environment effect models. Step 4 derives all possible combinations of the transitions affecting *p*. Step 5 calculates the total impact to *p* produced by each of the combinations. Step 6 examines if the total impact can trigger *E_S_B__* or not. Step 7 derives the pre-condition of the service chain. Finally, Step 8 validates if the pre-condition does not contradicts to the enabling condition of *S_A_*.

In our service chain detection method, the conditions under which the potential conflicts may arise are derived from static structures of service models. Since our method does not need to utilize an exhaustive search such as model checking, the state explosion problem does not occur if service complexity increases.

### 4.2. Running Example

Using the proposed algorithm, we demonstrate to detect the service chain C1 (DVD-T⇒ALC) in Section 1. In this example, we use the service description in Figure 1, and the environment effect model of the appliances in Table 1.

#### 4.2.1. Detection of Service Chain (DVD-T ⇒ ALC)

In Step 1, we pick up env.brightness from *E_ALC_* and know that the brightness is the key property of the service chain. In Step 2, we obtain methods TV.on(), Light.setbrightness(1) from *A_DVD_*_–_*_T_*, since they have effects on env.brightness. In Step 3, we identify the corresponding transitions in the environment effect models. In Table 1(b), we find *T*_*TV.on*()_ = { (OFF,on(),ON),(ON,on(),ON)}. In Table 1(c), we find *T_Light.setbrightness_*_(1)_ = { (OFF, setBrightness(1), ON), (ON, setBrightness(1), ON)}. Step 4 constructs a product *T_TV.on_*_()_ × *T_Ligh.setbrightness_*_(1)_ to derive the following four transition sequences.

- *tt*_1_ = ((OFF,on(),ON),(OFF,setBrightness(1),ON))
- *tt*_2_ = ((OFF,on(),ON),(ON,setBrightness(1),ON))
- *tt*_3_ = ((ON,on(),ON),(OFF,setBrightness(1),ON))
- *tt*_4_ = ((ON,on(),ON),(ON,setBrightness(1),ON))

Step 5 calculates the total the environment effect to env.brightness. Since the brightness is cumulative, we follow the Semantics S1.

- *TE*(*tt*_1_) = +200 + 100 = +300
- *TE*(*tt*_2_) = +200 +100 (1−bLevel)=300 − 100 * bLevel
- *TE*(*tt*_3_) = + 0 + 100 = +100
- *TE*(*tt*_4_) = + 0 + 100(1 − bLevel)=100 − 100 * bLevel

In Step 6, let *s_d_* be any state where *ALC* is not triggered, *i.e.*, ¬*E_ALC_* = env.brightness ≥ 200 holds. Also, let *s_e_* be any state where *ALC* is triggered, *i.e., E_ALC_* = env.brightness < 200 holds. Among *tt*_1_ to *tt*_4_, we want to find any transition sequences that can change *s_d_* to *s_e_*. Since both *tt*_1_ and *tt*_3_ give positive impact to the brightness, they cannot move *s_d_* to *s_e_*. So, *tt*_2_ and *tt*_4_ are chosen as the potential transition sequences of the service chain.

In Step 7, we first derive the pre-condition of the service chain from *tt*_2_. In order for the sequence *tt*_2_ to be executed, the TV must be OFF and the light is ON beforehand. So, *dcond_bright_* = [TV is OFF ∧ Light is ON]. From ¬*E_ALC_*, we obtain a condition env.brightness ≥ 200 to be satisfied *before* DVD-T. Also, as for the condition *after* DVD-T, we have env.brightness +*TE*(*tt*_2_) < 200 so that ALC is triggered. Therefore, *econd_bright_* = [200 ≤ env.brightness < 100 * bLevel−100]. Similarly, we derive the pre-condition from *tt*_4_. As a result, we can see that the service chain DVD-T⇒ALC occurs when DVD-T is executed under the one of the following pre-conditions.

- cond1: TV is OFF, Light is ON and a condition [200 ≤ env.brightness < 100 * bLevel−100] holds, or
- cond2: TV is ON, Light is ON and a condition [200 ≤ env.brightness < 100 * bLevel+100] holds.

These pre-conditions are validated in Step 8 that they do not disable DVD-T. So, we finally detect [ cond1] DVD-T⇒ALC and [ cond2] DVD-T⇒ALC.

## 5. Estimation of the Severity of a Service Chain Using Prior Expectation of Sensor-Driven Service

### 5.1. Severity of a Service Chain

As described in Section 3.4, every service chain does not lead to harmful feature interactions. Then, we define the degree of severity of a service chain, in order to evaluate whether the service chain is harmful. Intuitively, we consider that the service chain is serious, when the state after a service execution differs from the state after a service chain significantly We define a set *es* = (*v*_1_, *v*_2_, …, *v_n_*) of arbitrary value of environment properties *p*_1_, *p*_2_, …, *p_n_* as an environmental state. When an environmental state *es*[*S_A_*] at the time of *S_A_* being executed independently and other environmental state of *es*[*S_A_* ⇒ *S_B_*] at the time of a service chain *es*[*S_A_* ⇒ *S_B_*] being executed are compared, we estimate that the gap between both environmental states is large.

### 5.2. Prior Expectation in a Sensor-Driven Service

It is also dependent on the requirement(*prior expectation*) which a user expects to be satisfied by a service whether the service chain is serious or not. The prior expectation is expressed as a state of appliances and environment properties contained in the main purpose of a service. Table 2 shows examples of prior expectation in each service. In the table, the prior expectation in ARH is related to “indoor temperature”. Similarly, the prior expectation in ESAC is related to “electricity”. Therefore, when evaluating the degree of severity of a service chain, it is necessary to narrow down a state *es* in the view point of the prior expectation.

We define a projection *es* = (*v*_1_, *v*_2_, …, *v_n_*) to *I* = {*p_i_*_1_, …, *p_ik_*}(a set of environmental properties) as *proj_I_*(*es*) = (*v_i_*_1_, …, *v_ik_*). Let *I_A_* be a set of environment properties related to the prior expectation in *S_A_*. Moreover, let *es*[*S_A_*] be a state at the time of *S_A_* being executed independently, and *es*[*S_A_* ⇒ *S_B_*] be a state at the time of *S_A_* ⇒ *S_B_* being executed. Here, *proj_I_A__*(*es*[*S_A_*]) can be considered as a prior expectation expected by a user to *S_A_*. Similarly, *proj_I_A__*(*es*[*S_A_* ⇒ *S_B_*]) is regarded as an actual state at the time of *S_A_* ⇒ *S_B_* being executed.

### 5.3. Estimation of Service Chain Severity

We estimate whether a service chain causes serious feature interaction or not, depending on whether the difference between *proj_I_A__*(*es*[*S_A_*]) which is expected by a user to *S_A_* and *proj_I_A__*(*es*[*S_A_*]) is below a threshold value *τ*. Here, *τ* is a given value experientially by a user or the service creator.

#### Assumption 2

A sensor-driven service *S_A_* = (*E_S_A__, C_S_A__, A_S_A__*) and *S_B_* = (*E_S_B__, C_S_B__, A_S_B__*) cause a service chain [*cond*]*S_A_* ⇒ *S_B_*. A set of properties which a user expects to the *S_A_* is *I_A_* = {*p_i_*_1_, …, *p_ik_*}.

Each *p_ij_* is given a threshold value *τ*(*p_ij_*).

**Step 1:** Create a set of state *es* = (*v*_1_, *v*_2_, …, *v_n_*) which is formed by the *cond*.
**Step 2:** In the *es*, the method of *A_S_A__* is virtually invoked according to each appliance model. Let *tt_A_* = (*t*_1_, *t*_2_, …, *t_s_*) be a set of transition sequence of the appliance model performed. We calculate $es\left[S_{A}\right]=\left(v_{1}^{\prime },v_{2}^{\prime },\ldots ,v_{n}^{\prime }\right)$ by compounding the total environment effect *TE*(*tt_A_*)(described in Section 4.1) to *es.*
**Step 3:** In the *es, A_S_A__* and *A_S_B__* are invoked in order. Let *tt_A,B_* = (*t*_1_, *t*_2_, …, *t_l_*) be a set of transition sequence performed in the service chain. We calculate $es\left[S_{A}\Rightarrow S_{B}\right]=\left(v_{1}^{*},v_{2}^{*},\ldots ,v_{n}^{*}\right)$ by compounding the total environment effect *TE*(*tt_A,B_*) to each element in *es*.
**Step 4:** Calculate the gap between a state caused by prior expectation and an actual state by the service chain as follows. ${\text{proj}}_{I_{A}}\left(\left[S_{A}\right]\right)-{\text{proj}}_{I_{A}}\left(\left[S_{A}\Rightarrow S_{B}\right]\right)=\left(v_{i1}^{\prime },v_{i2}^{\prime },\ldots ,v_{ik}^{\prime }\right)-\left(v_{i1}^{*},v_{i2}^{*},\ldots ,v_{ik}^{*}\right)=\left(v_{i1}^{\prime }-v_{i1}^{*},v_{i2}^{\prime }-v_{i2}^{*},\ldots ,v_{ik}-v_{ik}^{*}\right)$
**Step 5:** Determine *S_A_* ⇒ *S_B_* as a serious service chain if $\left|v_{ij}^{\prime }-v_{ij}^{*}\right|>\tau \left(p_{ij}\right)\;\left(1\leq j\leq k\right)$ is true.

Here, if a service has multiple environment properties as the target of prior expectation, our algorithm calculates each severity separately. For instance, if a service has brightness property and temperature property as the target of prior expectation, severities of the service in the view point of brightness and temperature are calculated respectively, and the service is judged whether the service chain is a feature interaction or not.

Second, if a service does not include any prior expectation, it is difficult to capture the user's desire about the service. We assume the prior expectation depends on user's requirement or desire. Therefore, in our future approach, though the service chain is detected, the severity of the service is not calculated.

Our method to estimate the severity of service chains generates a set of state which satisfies the conditions. Though the set of state which satisfies the conditions may exist infinitely, environment properties not included in prior expectations are treated symbolically in our method. For example, a state about a service which includes brightness property as the prior expectation is expressed like (Temperature, Brightness, Electricity) = (x, 800, y) in internal implementation. An environment property included in a prior expectation may take various values. Two or more typical values should be checked for estimating severity. The typical values should reflect user's desire or some kinds of situation in which the service is invoked. Concrete methods to configure the property values for severity estimation are left for our future research.

### 5.4. Running Example

We evaluated the degree of severity about the service chain in Section 1. Here, we focused on three properties, such as temperature, brightness and electricity in HNS. Moreover, the threshold of deviation *tau* is 2 (°C), 500 (lx), and 500 (W), respectively.

#### 5.4.1. Severity Evaluation of DVD-T⇒ALC

In Step 1, we create an environmental state *es* = (temperature, brightness, electricity)= (24, 800, 50) which may trigger the service chain. Here, current brightness setting of the light in the services is level 8. In Step 2, when only DVD-T is invoked, *es*[*DVD* − *T*] changes to (24, 100, 850). In Step 3, when DVD-T and ALC are invoked in order, *es*[*DVD* − *T* ⇒ *ALC*] changes to (24, 1000, 850). In Step 4, if *I_DVD_*_−_*_T_* is {*brightness*}, the gap is calculated as follows. *proj_I_DVD−T__*(*es*[*DVD* − *T*]) — *proj_I_DVD−T__*(*es*[*DVD* − *T*] ⇒ *ALC*) = (100) − (1000)= (−900) That is, user's prior expectation of brightness in DVD-T has significant deviation: −900 from the actual environment state by the service chain. In Step 5, |−900| > *τ*(brightness) = 500. As a result, this service chain can be considered serious.

## 6. Implementation of Service Chain/feature Interaction Detection System

### 6.1. Architecture

**Figure 2.**
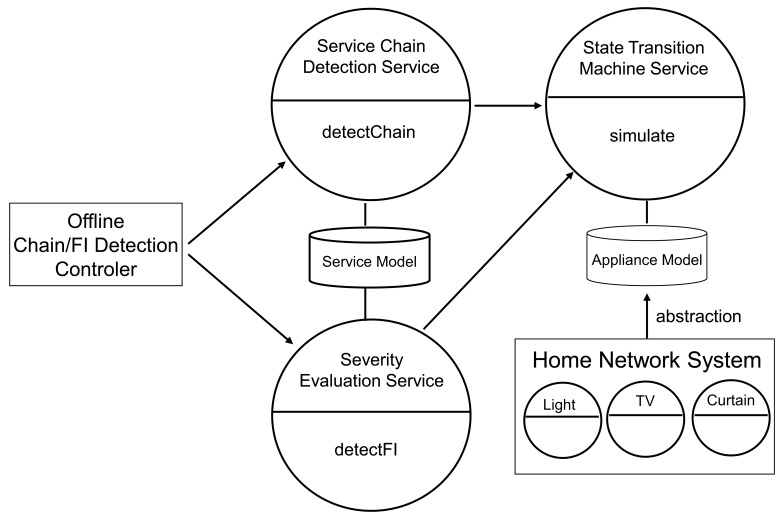
Service chain/FI detection system Architecture.

Figure 2 shows the architecture of our service chain/feature interaction detection system. Our system consists of *a state transition machine service, a service chain detection service, a severity evaluation service* and *an offline service chain/feature interaction detection controller*. The chain detection controller detects service chains and feature interactions by invoking the service chain detection service and the severity evaluation service. Henceforth, the details of each component are explained.

#### 6.1.1. State Transition Machine Service

The state transition machine service calculates the environment effect *e*(*t*) for each appliance based on the appliance model.

This service exhibits the following methods.

**imulate(String[] methods):SimulationResult[]** This method receives an array of appliance method (String type) as an argument, and returns an array of the SimulationResult class. This method returns SimulationResult class which contains combination of the environment effect and the appliance state transition based on the given appliance method sequence. The SimulationResult has the following attributes.
**SimulationResult**
  - effects(Effect[]) : An array of the environment effect for each environment property
  - transitions(Transition[]) : An array of appliance transition sequence caused by appliance method execution
**Effect**
  - propertyName(String) : A name of an environment property
  - value(double) : Degree of environment effect
  - operator(String) : Operator is any one of ”=”,”+=”,”-=”.
**Transition**
  - deviceName(String) : Device name
  - preState(String) : A state before transition
  - postState(String) : A state after transition

This method controls Steps 2–5 in Section 4.1 and Steps 2 and 3 in Section 5.3.

#### 6.1.2. Service Chain Detection Service

The service chain detection service stores the service models and detects a service chain between given sensor-driven services. This service exhibits the following method.

**detectChain(String service1, String service2):boolean** This method receives names of two sensor-driven services (a source service and a destination service), which may cause a chain reaction, as arguments, and returns a boolean value which indicates whether there exists a service chain. This method controls our service chain detection algorithm in 4.1. First, this method gets an action of the source service and an event of the destination service from the service models. Next, this method calculates a total environment effect of the source service with using the simulate() method in the state transition machine service.

#### 6.1.3. Severity Evaluation Service

The severity evaluation service stores service models, and evaluates degree of a severity of a given service chain. This service exhibits the following method.

**detectFI(String service1, String service2):boolean** This method receives names of two sensor-driven services (a source service and a destination service), which may cause a chain reaction, as arguments, and returns a boolean value which indicates whether there exists a feature interaction. This method controls our severity evaluation algorithm in Section 5.3. First, this method gets method information in the service model. Next, this method calculates an environment state after execution of the source service, and another environment state after the occurrence of the service chain, respectively. Then, this method calculates the degree of deviation of the environment state for every property which is related to the prior expectation in the source service which may cause the chain reaction.

### 6.2. Implementation and Evaluation

The total lines of code of our program are about 4,300, and the programming language used for development is Java. We use the actual HNS(CS27-HNS) developed by our research group for sensor-driven services. All the services included in the service chain/feature interaction detection system were deployed under the following environments.

- Server Specification: 950 MB RAM 2.00 GHz WinXP Pro
- Tomcat 5.5
- Apache Axis 2.2
- Java JDK5

We performed the service chain/feature interaction detection for the 7 services in Figure 1. Our system required about 16 seconds for detecting the service chains and the feature interactions.

## 7. Case Study

To evaluate the proposed method, we have conducted a case study to detect all service chains and feature interactions among the seven sensor-driven services shown in Figure 1.

Table 3 shows the result. In the table, a row represents a service triggered first (called first service, say *S_A_*), and a column represents one triggered second (called second service, say *S_B_*). Each entry shows whether a service chain *S_A_* ⇒ *S_B_* occurs or not. Out of all 42(= 7×6) possible combinations, 11 service chains and 6 feature interactions were automatically detected by our system (marked as Chain and FI in Table 3). Scenarios of DVD-T⇒ALC and ARH⇒ESAC are the same as C1 and C2 in Section 1, respectively. Scenarios of other FIs are explained below.

### 7.1. Service Chain Scenario (ESIA⇒ARH)

In a day of winter, as nobody remains the room, ESIA turns off all the appliances. This situation makes the temperature decrease, and ARH is triggered in due course. The service chain violates the goal of energy-saving of ESIA.

### 7.2. Service Chain Scenario (LH⇒ARH)

This service chain is similar to ESIA⇒ARH. As a user presses the button of LH, all the appliances are shut down. This makes the temperature decrease and has ARH triggered. This service chain is against the user's requirement that all the appliances should be off while leaving home.

### 7.3. Service Chain Scenario (ARH⇒ARC)

As the temperature decreases, ARH is triggered to heat the room using the air-conditioner. This makes room warm enough to trigger ARC to cool the room, which violates the goal of ARH. The algorithm derives a pre-condition that the air-conditioner is OFF or working with the temperature setting 25 °C or less.

### 7.4. Service Chain Scenario (LH⇒ALC)

If a user presses the button of LH in the night, all the appliances are shut down, and the room becomes dark. Then, ALC is triggered and the light is turned on again. The chain violates the user's requirement that all the appliances should be off while leaving home. The algorithm derives a pre-condition that [200 ≤ env.brightness < 200 + 100* Light.bLevel, the light is ON, and TV is OFF].

## 8. Discussion

### 8.1. Advantage and Limitations

For given sensor-driven services, the proposed method can detect all potential service chains and feature interactions automatically. It also derives concrete pre-conditions for every service chain detected. Therefore, the proposed method can make service developers aware of unexpected service chains in advance. Furthermore, we could calculate the degree of severity of each service chain and judge the undesirable feature interactions resulting from the service chain.

In this sense, the proposed method is useful especially for the offline validation at the design stage of the services.

On the other hand, the detected harmful service chain needs to be resolved. However, this paper does not show the concrete resolution method for such service chains. It is necessary to define the suitable resolution method according to the degree of severity of the service chain. A service developer and a user are to determine the magnitude of the deviation experientially. More systematic determination method for the deviation is our future subject.

It is significant problem to determine adequate value of the *τ*. The value of *τ* is a criterion which indicates the degree of the severity of detected feature interactions. Accordingly, wrong value of *τ* does not capture the user's desire. On the other hand, since the value of *τ* depends on user's requirement, desire, or environment where sensor-driven services are deployed, it is significantly difficult to decide the value statically in advance. Currently, a method to configure the value of *τ* and resolve feature interactions are both left for our future research. In our next research, we consider about a method to use the value small enough as an initial value of *τ*. In the approach, feature interactions are detected based on the initial value of *τ*, and a user decides and conveys whether each feature interaction is acceptable or not. The recursive feedback process between the user and our system make the value of *τ* configured gradually.

### 8.2. Related Work

Kolberg *et al.* [7] presented a classification of feature interactions in smart home, where our service chains can be categorized as the *sequential action interaction* (SAI). However, the concrete detection method of the service chains was not described. Wilson *et al.* [8] took the environment effects of the appliances for detecting feature interactions. However, since the amount of the effects was not explicitly considered, it would be difficult to derive detailed pre-conditions of the service chains. The ECA rules have been often used for detecting policy conflicts [6,9,10]. These methods basically focused on action conflicts only, and the service chains were not explicitly considered. In [11], we proposed a service-oriented framework that facilitates development of individual sensor-driven services. However, this method did not cover interactions among multiple services.

In distributed system, many techniques for detecting policy conflict based on policies such as Authorization Policy and Obligation policy have been proposed. Lupu *et al.* [12] have classified policy conflicts occurring in distributed systems and proposed conflict resolution method according to the classification. Dunlop *et al.* [13] have proposed dynamic and scalable policy conflict detection method. In the method, conflict DB stores the combination of the policies which may cause conflicts.

In the viewpoint of conflict detection, these researches differ from our proposed method detecting conflicts based on the service chain using the HNS component model. On the other hand, it is significant to apply the dynamic and scalable conflict detection method to our detection technique.

Charalambides *et al.* [14] have proposed conflict analysis using Event Calculus in the domain of QoS management. In the analysis method, they used abductive reasoning to detect the potential conflicts. Furthermore, they have identified a number of potential conflicts, and have classified them. In the classification, the redundancy conflict and the mutual exclusion conflict can be considered as some kinds of feature interactions in HNS [4] However, in this paper, we proposed FI detection method based on the service chain which differs from conventional FIs such as redundancy and mutual exclusion.

Shankar *et al.* [15] have proposed a method to detect conflicts and cycles in policies based on their rule framework “Event-Condition-Precondition-Action-Postcondition” in pervasive systems. Their conflict detection method using the rule framework ECPAP resembles our proposed method. However, since they assume that the framework is used in general-purpose pervasive systems, the subjects peculiar to the sensor-driven services in HNS have not necessarily been solved. We have proposed our framework which takes multiple different kinds of environment properties into account. Furthermore, our framework provides the concrete conditions under which each potential conflict may arise and estimation of severity of the conflicts quantitatively.

## 9. Conclusions

In this paper, we have proposed a method that detects service chains among sensor-driven services in the home network system (HNS), and evaluates the severity of the detected service chains. The proposed method adopts the ECA rules for the service description, and introduces the environment effect model to define the effect of the appliances to the environment, explicitly. We also implemented the proposed method as a tool and detected 11 service chains and 6 feature interactions among 7 practical services. Based on our method, we proposed systematic resolution schemes of undesirable service chains. Extending the method for general sensor services outside the HNS is also a challenging issue.

## Figures and Tables

**Table 1.** Environment Effect Model. (**a**) Air-Conditioner; (**b**) TV; (**c**) Light.

		**Method**		
	
		**off()**	**heating(setTemp)**	**cooling(setTemp)**
State	OFF	next : OFF	env. electricity += 1500	env. electricity += 1500
			env. temperature = setTemp	env. temperature = setTemp
			next : HEATING	next : COOLING

	HEATING	env.electricity -= 1500	env. temperature = setTemp	env.temperature = setTemp
		env.temperature =10	next : HEATING	next : COOLING
		next : OFF		

	COOLING	env.electricity -= 1500	env.temperature = setTemp	env.temperature = setTemp
		env.temperature =10	next : HEATING	next : COOLING
		next : OFF		

(**a**)				

		**Method**	
	
		**off()**	**on()**
State	OFF	next : OFF	env.electricity += 500
			envbrightness += 200
			next : ON

	ON	electricity -= 500	next : ON
		env.brightness -= 200	
		next : OFF	

(**b**)			

		**Method**		
	
		**off()**	**on()**	**setBrightness(setB)**
State		next : OFF	env.brightness +=	env.brightness += 100 * setB
	OFF		100 * this.bLevel	env.electricity +=50
			env.electricity +=50	this.bLevel = setB
			next : ON	next : ON

		env.brightness -=	next : ON	env.brightness +=
	ON	100 * this.bLevel		100 *(setB-this.bLevel)
		env.electricity -=50		this.bLevel = setB
		next : OFF		next : ON

(**c**)				

**Table 2.** Service prior expectations.

		Environment Property		
		temperature	brightness	electricity
Service	DVD-T		○	
	ALC		○	
	ARH	○		
	ESAC			○
	ARC	○		
	LH			○
	ESIA			○

**Table 3.** Service chain/Feature Interaction detection result.

		Second Service S_B_						
		DVD-T	ALC	ARH	ESAC	ARC	LH	ESIA
First Service S_A_	DVD-T		Chain FI		Chain			
	ALC				Chain			
	ARH				Chain FI	Chain FI		
	ESAC			Chain				
	ARC				Chain			
	LH		Chain FI	Chain FI				
	ESIA		Chain	Chain FI				
